# Fibulin 2 Is Hypermethylated and Suppresses Tumor Cell Proliferation through Inhibition of Cell Adhesion and Extracellular Matrix Genes in Non-Small Cell Lung Cancer

**DOI:** 10.3390/ijms222111834

**Published:** 2021-10-31

**Authors:** Yunxia Ma, Miljana Nenkov, Desiree Charlotte Schröder, Mohamed Abubrig, Nikolaus Gassler, Yuan Chen

**Affiliations:** Section Pathology of the Institute of Forensic Medicine, Jena University Hospital, Friedrich Schiller University Jena, Am Klinikum 1, 07747 Jena, Germany; yunxia.ma@med.uni-jena.de (Y.M.); miljana.nenkov@med.uni-jena.de (M.N.); desiree.schroeder97@gmail.com (D.C.S.); Mohamed.abubrig@med.uni-jena.de (M.A.); Nikolaus.gassler@med.uni-jena.de (N.G.)

**Keywords:** NSCLC, FBLN2, DNA methylation, cell adhesion, ECM

## Abstract

Fibulins (FBLNs), interacting with cell adhesion receptors and extracellular matrix (ECM) components, play multiple roles in ECM structures and tissue functions. Abnormal expression of FBLN2, one of the fibulin family members, contributes to tumor initiation and development. However, the function of FBLN2 in human non-small cell lung cancer (NSCLC) has not yet been elucidated. In this study, we found that FBLN2 was downregulated in 9 out of 11 lung cancer cell lines compared to normal bronchial epithelial cells, which was associated with DNA hypermethylation. Primary lung squamous cell carcinoma expressed significantly more FBLN2 protein compared to adenocarcinoma (*p* = 0.047). Ectopic expression of FBLN2 led to decreased cell proliferation, migration and invasion, accompanied by inactivated MAPK/ERK and AKT/mTOR pathways, while FBLN2 siRNA knockdown resulted in an opposite biological behaviour in NSCLC cells. Additionally, overexpression of FBLN2 led to dysregulation of cell adhesion molecules, ECM markers and a panel of lysate/exosome-derived-microRNAs, which are involved in cell adhesion and ECM remodelling. Taken together, our data indicate that FBLN2 is methylated and exerts a tumor suppressor function through modulation of MAPK/ERK and AKT pathways and regulation of cell adhesion and ECM genes. Moreover, FBLN2 might be a potential biomarker for the sub-classification of NSCLC.

## 1. Introduction

The Human fibulin family, as one of the participators in elastic fiber assembly, is closely associated with basement membrane integrity, and is involved in multiple cellular processes, including cell shape, motility, and growth, that are crucial for the regulation of tissue morphogenesis [1,2]. Fibulin 2 (FBLN2) is the second largest molecule of the fibulin family, and it is localized at the interface between elastin cores and microfibrils [3]. According to the database of the Human Protein Atlas [4], FBLN2 is highly expressed in female tissue, muscle tissue, and the brain, and moderately expressed in the lungs, skin, and tonsils.

As an extracellular glycoprotein, Fibulin 2 binds to and interacts with multiple extracellular matrix (ECM) components to assemble and stabilize ECM. In a previous study, single knockout mice for fbln2 were found to be viable and fertile and did not show obvious anatomical abnormalities, indicating that fbln2 might be dispensable for mouse development due to a compensatory mechanism that upregulates other fibulin family members, such as fbln1 [5] and fbln5 [6]. However, severe disruption of the elastic lamina has been observed in double knockout mice for fbln2 and fbln5, suggesting that fbln2 and fbln5 may cooperatively contribute to the formation of ECM [6]. Additionally, FBLN2 is associated with the outgrowth of pubertal and early pregnant mouse mammary epithelium, and loss of FBLN2 is related to a disrupted basement membrane integrity and early invasion of mouse mammary epithelial cells [7,8]. Additionally, FBLN2 binding to cell adhesion molecules, such as integrin subunits, further strengthens the stability of ECM [9,10], and modulates ECM-mediated extracellular–intracellular signalling regulation through integrins [11].

The Fibulin 2 protein has a multidomain structure that contains a cys-rich domain, three anaphylatoxin modules, a tandem of epidermal growth factor (EGF)-like domains, and a fibulin-like domain [12]. Two isoforms have been identified in Fibulin 2. The long form consists of 1231 amino acids, and the short form, lacking the third EGF-like domain (aa709–755), comprises 1184 amino acid [13]. Both isoforms are present in human and mice, but their functional difference is not well determined [14]. It has been reported that the short form of FBLN2 is dominantly expressed in human nasopharyngeal carcinoma [15], breast, colon [16] and lung cancer [17].

Beyond its structural role, FBLN2 participates in carcinogenesis through regulating the composition of ECM to influence the stabilization and integrity of the basement membrane. FBLN2 interacts with MUC4, leading to a breach of the basement membrane (BM), which promotes the invasion of pancreatic cancer [18]. In nasopharyngeal cancer, FBLN2 inhibits the metastatic properties via downregulation of vascular endothelial growth factor (VEGF) and matrix metalloproteinase 2 (MMP2), one of the ECM associated enzymes [15]. Downregulation of β-catenin, a subunit of the cadherin protein complex and a signal transducer of the Wnt pathway, has been found to be involved in the antitumor effect of FBLN2 in gastric cancer [19]. Additionally, FBLN2 is considered a favorable prognostic marker in liver cancer but an unfavorable marker in endometrial cancer [20]. Recently, it was found that FBLN2 was significantly more expressed in the plasma of patients with meningioma grade II, compared to patients with grade I meningioma, suggesting that FBLN2 might be a non-invasive biomarker for this disease [21]. In human lung adenocarcinoma, FBLN2 shows both ‘fibrillar’ and ‘fibrotic’ expression patterns, indicating that FBLN2 might have multiple cellular origins and biological functions [22]. However, there is limited knowledge about the function of FBLN2 in human lung cancer, and the regulatory role of FBNL2 in cell adhesion and ECM construction in human lung cancer is also largely unknown.

MicroRNAs (miRNAs or miRs) such as miR-9, miR-21, miR-29, miR-199 miR-200 and miR-155 are involved in the regulation of cell–cell adhesion and ECM remodeling [23]. Current research data have not only revealed that miRNAs can modulate the cell adhesion and composition of the ECM, but also that cell adhesion and the ECM can influence the expression of specific miRNAs [24]. For example, in fbln4 KD mice with ECM defects, the expression of miR-29 was significantly elevated in the aortic arch; further inhibition of miR-29 upregulated the expression of ECM components including COL1A1, COL3A1 and Elastin [25]. Therefore, it could be interesting to analyze cell adhesion, the ECM, and miRNA expression in a FBLN2 context.

Thus, in this study, we investigated the function and the epigenetic regulation of FBNL2 in NSCLC, and additionally, the regulatory role of FBNL2 in cell adhesion molecules, ECM components, and their key modulators, particularly miRNAs, was explored. Furthermore, the clinical significance of FBNL2 in NSCLC was evaluated.

## 2. Results

### 2.1. FBLN2 Is Downregulated in Lung Cancer

**Figure 1:**
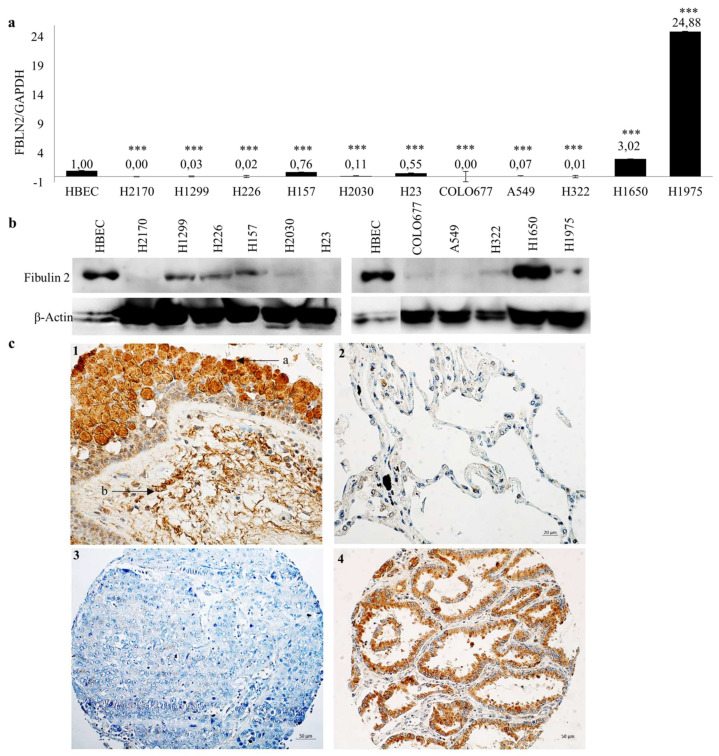
FBLN2 expression in human lung cancer cell lines, normal lung tissue and primary lung tumors. FBLN2 expression in HBEC and lung cancer cell lines was determined by using real-time RT-PCR (**a**) and WB (**b**). The FBLN2 mRNA expression level compared to the expression level of the internal control glyceraldehyde 3-phosphate dehydrogenase (GAPDH) in HBECs was defined as 1.0, *** *p* < 0.001. β-actin was the loading control for WB; (**c**) Representative immunohistochemical staining of fibulin 2 in normal lung tissue and primary lung tumors. 1-a: Bronchial epithelial cells, 1-b: Stroma tissues. Magnification: 400×. Scale bar: 20 µm. 2: Alveoli. Magnification: 400×. Scale bar: 20 µm. 3: SCC, score 0. Magnification: 200×. Scale bar: 50 µm. 4: ADC, score 3. Magnification: 200×. Scale bar: 50 µm.

FBLN2 isoforms exhibit a dramatic expression shift during tumorigenesis in nine different cancers, including lung cancer [15,17]. To analyse the expression of FBLN2 long and short isoforms (without exon 9), RT-PCR was performed in normal human brachial epithelial cells (HBEC) and a panel of lung cancer cell lines. It was found that the short isoform is the dominant isoform in the majority of lung cancer cells, while in HBEC, both isoforms were present (Supplementary Figure S1). Real-time RT-PCR and Western blotting (WB) were performed to further confirm the expression pattern of FBLN2. Compared to HBEC, FBLN2 was downregulated in 9 or 10 out of 11 lung cancer cell lines on the mRNA or protein level (Figure 1a,b).

In the lung tissues with normal histology adjacent to the tumors, fibulin 2 is highly expressed in bronchial epithelial cells and in the stroma tissues (Figure 1c), however, the alveoli display very low or even no expression of fibulin 2. In primary tumor tissues, positive staining of fibulin 2 was found in 46.2% (24/52) of adenocarcinoma (ADC) and 66.7% (28/42) of squamous cell carcinoma (SCC), and this difference was statistically significant (Figure 1c and Table 1). No significant association was found between fibulin 2 expression and gender, tumor stages N/M, tumor grade as well clinical outcome, whereas a tendency was detected between a higher expression of fibulin 2 and a smaller tumor size (*p* = 0.061), suggesting that fibulin 2-positive expression may be related to early tumor stages. 

### 2.2. Epigenetic Regulation Contributes to the Downregulation of FBLN2

**Figure 2:**
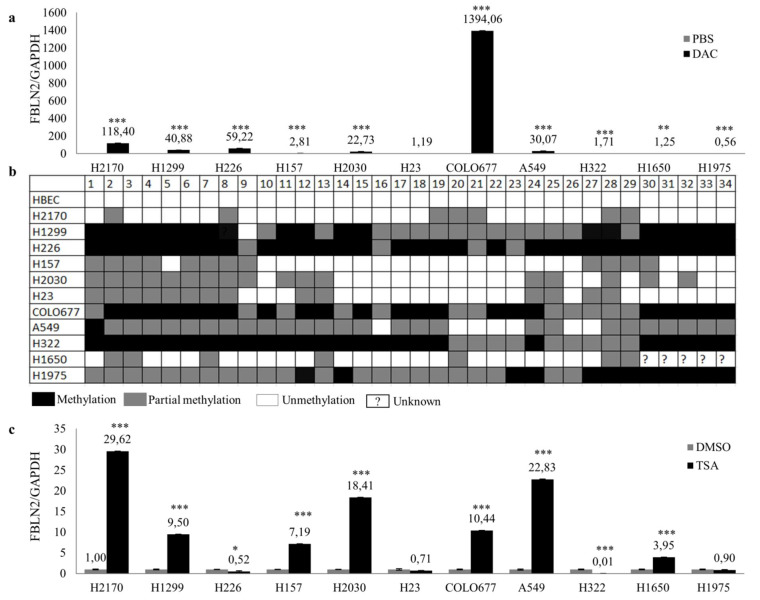
Epigenetic regulation of FBLN2 in normal human bronchial epithelial cells and lung cancer cell lines. (**a**) mRNA expression of FBLN2 was measured by real-time RT-PCR after demethylation treatment with 5 µM of 5-aza-2′-deoxycytidine (DAC); (**b**) Methylation status of FBLN2 in the promoter region was analyzed by using bisulfite sequencing; (**c**) mRNA expression of FBLN2 was analyzed after the cells were treated with the histone deacetylase inhibitor Trichostatin A (TSA). mRNA expression level compared to GAPDH in untreated cells was set to 1.0. * *p* < 0.05, ** *p* < 0.01, *** *p* < 0.001.

Promoter hypermethylation is responsible for FBLN2 silencing in breast cancer and nasopharyngeal cancer [15,26]. To explore whether DNA methylation plays a role in FBLN2 downregulation in lung cancer cell lines, cells were treated with 5-aza-2′-deoxycytidine (DAC), a demethylation agent, and then real-time RT-PCR and WB were performed. FBLN2 was widely restored in all lung cancer cell lines on the mRNA level except H23 and H1975 (Figure 2a) and increased in H157, H2030 and A549 on the protein level (Supplementary Figure S2). Furthermore, the bisulfite sequencing and methylation-specific-PCR (MSP) were applied to analyze the methylation status of FBLN2 promoter in both lung cancer cell lines and tumor tissues. A variable FBLN2 DNA methylation was found in lung cancer cell lines, whereas no methylation of FBLN2 DNA was detected in control HBEC (Figure 2b and Figure S3). DNA methylation of FBLN2 in lung cancer cell lines was confirmed by MSP, confirming the utility of MSP for the analysis of the methylation status of FBLN2 in tissue samples (Supplementary Figure S4). In primary lung tumor tissues, DNA methylation of FBLN2 was detected in 81.1% (30/37) of tumor tissues. However, we did not find a significant correlation between DNA methylation and Fibulin 2 protein expression (Table 1). Additionally, FBLN2 mRNA expression was restored in all lung cell lines except H226, H23, H322 and H1975 after treatment with the histone deacetylase inhibitor Trichostatin A (TSA) (Figure 2c).

Taken together, our data suggest that downregulation of FBLN2 in lung cancer cell lines may be associated with DNA methylation and histone acetylation. 

### 2.3. FBLN2 Suppresses Cell Colony Formation, Invasion and Migration through Inhibiting the MAPK/ERK and PI3K/AKT/mTOR Pathways

**Figure 3:**
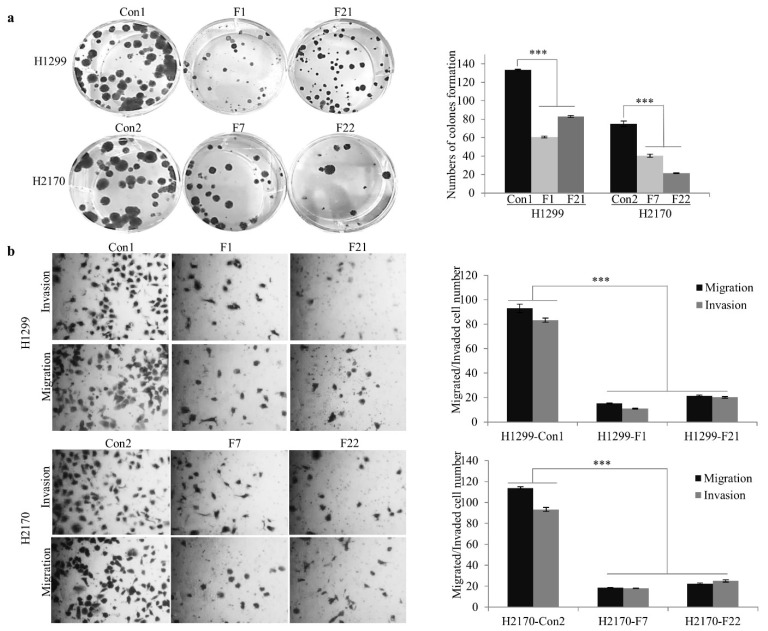
Impact of FBLN2 on NSCLC cell colony formation, migration and invasion. (**a**) Colony formation assay showed that FBLN2 inhibited NSCLC cell proliferation. (**b**) Reduced migration and invasion capabilities by FBLN2 were determined by migration and invasion assays. Left: Representative images of the colony formation, migration and invasion assays. Right: Quantification of the cell numbers of the assays. *** *p* < 0.001.

To further investigate the biological role of FBLN2, H1299 (ADC) and H2170 (SCC) cells were transfected with an expression vector containing the FBLN2 short isoform. Stable expression of FBLN2 was confirmed on the mRNA and protein levels (Supplementary Figure S5). Two positive colonies (F1 and F21 for H1299; F7 and F22 for H2170) were applied for the cell-based functional assays. Overexpression of FBLN2 extremely reduced colony formation by 53.8% and 41.1% in H1299 and H2170, respectively (Figure 3a). In addition, FBLN2 considerably reduced the migrated and invaded cell numbers in FBLN2-transfected cells in both H1299 and H2170 (Figure 3b). Altogether, these results suggest that FBLN2 has the potential to inhibit lung cancer cell proliferation.

**Figure 4:**
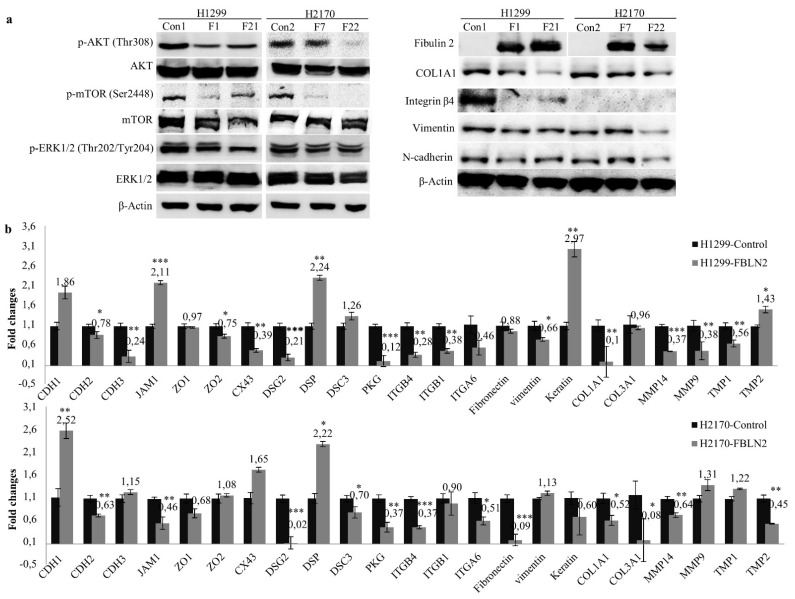
Influence of FBLN2 on regulation of MAPK and AKT pathways, cell adhesion molecules, and ECM associated genes. (**a**) Left: Reduced phosphorylated levels of ERK1/2, AKT and mTOR in FBLN2 transfected cells were determined by WB. Right: Decreased protein levels of cell adhesion molecules and ECM components in FBLN2 overexpressing cells were detected by WB. (**b**) A panel of cell adhesion molecules and ECM genes were measured by real-time RT-PCR in H1299 (top) and H2170 cells (bottom). mRNA expression level compared to GAPDH in mock cells was set to 1.0. * *p* < 0.05, ** *p* < 0.01, *** *p* < 0.001.

Mitogen activated protein kinase (MAPK) and phospho-inositide 3-kinase (PI3K)/AKT signaling pathways maintain constitutive activity during cancer cell growth [27]. To understand the underlying mechanism responsible for cell growth inhibition, MAPK and AKT signaling pathways were analyzed. We found that the ectopic expression of FBLN2 inhibited the activity of phosphorylated ERK1/2 and phosphorylated AKT, as well as that of phosphorylated mTOR (Figure 4a, left). In all, our data suggest that FBLN2 may suppress lung cancer cell proliferation through regulating MAPK/ERK and PI3K/AKT/mTOR pathways.

### 2.4. FBLN2 Suppresses Tumor Cell Proliferation by Regulation of Cell Adhesion Molecules and ECM Associated Genes

On the one hand, FBLNs, as extracellular, multidomain glycoproteins interact with multiple ECM components to assemble and stabilize the biological ECM scaffolds, and on the other hand, they bind to cell adhesion molecules to facilitate signal transduction [2]. To explore whether decreased motile and invasive properties by FBLN2 are associated with the alteration of cell adhesion molecules and ECM genes, we analysed the protein expression of the ECM components COL1A1 and ITGN4 (integrin β4), the intermediate filament protein vimentin, and the cell adhesion protein N-cadherin after FBLN2 overexpression by WB. Further, the genes involved in cell adhesion and the ECM, as listed in Supplementary Figure S6, were analysed by real-time RT-PCR. Our data showed that overexpression of FLBN2 led to obvious downregulated protein levels of COL1A1, integrin β4, vimentin, and N-cadherin in H1299 (ADC) (Figure 4a, right). In H2170 (SCC), decreased expression levels of COL1A1, vimentin and N-cadherin were detected in at least one FBLN2-positive clone (Figure 4a, right).

Additionally, upregulation of the tight-junction-associated gene JAM; the desmosomal gene DSP; keratin, one of the ECM scaffold molecules; and the ECM associated gene TMP2, and downregulation of the adherent-junction molecule CDH2, the tight-junction-associated gene ZO2, the gap-junction-related gene CX43, the desmosomal gene PKG, the cell adhesion receptor ITGB4 and the ECM components COL1A1 as well as the ECM-associated genes MMP14, MMP9, and TMP1, were found after FBLN2 transfection in H1299 cells (Figure 4b, top). In H2170 cells, the mRNA expression of the adherent junction molecule CDH1 and desmosomal gene DSP was significantly enhanced, while the desmosomal genes DSG2, DSC3, and PKG, and the cell adhesion receptors ITGB4 and ITGA6, as well as ECM genes such as fibronectin, COL1A1, COL3A1, MMP14, and TMP2 were markedly decreased in FBLN2-transfected cells compared to mock cells (Figure 4b, bottom). The distinct gene expression pattern between the ADC and SCC cells may have been related to the different cell context.

We also analyzed the correlation between Fibulin 2, fibronectin, E-cadherin, vimentin, and cytokeratin in primary lung tumor tissues. Pearson correlation coefficients indicated that, in line with the reduced mRNA expression in the FBLN2-transfected SCC cells, higher fibulin 2 protein expression was significantly correlated with lower Fibronectin expression in primary lung SCC patients with a lower T stage (*r* = −0.391, *p* = 0.048, Table 2). Moreover, higher fibulin 2 was significantly correlated with higher levels of E-cadherin and cytokeratin in SCC subtype (Table 3).

### 2.5. FBLN2 Knockdown Promotes Migratory and Invasive Phonotypes

**Figure 5:**
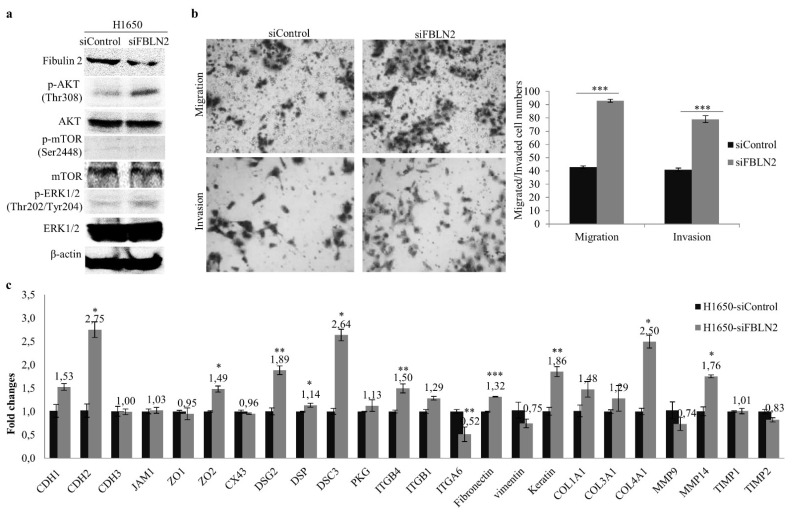
Effect of siRNA knockdown of FBLN2 on MAPK and AKT pathways and lung cancer cell migration/invasion. (**a**) Left: Downregulation of FBLN2 by siRNA-FBLN2 led to enhanced phosphorylation levels of ERK and AKT. (**b**) FBLN2 knockdown increased the cell migration/invasion. (**c**) FBLN2 gene silencing influenced the expression levels of cell adhesion and ECM genes, as revealed by real-time RT-PCR. mRNA expression level compared to GAPDH in cells transfected with siRNA-Control was set to 1.0. * *p* < 0.05, ** *p* < 0.01, *** *p* < 0.001.

To determine whether cells with downregulated FBLN2 display opposite behaviour and gene expression levels to the overexpression of FBLN2, siRNA-mediated interference of gene expression was conducted in the ADC cell line H1650 with the endogenous expression of FBLN2. Compared to the control, FBLN2 was downregulated on both the mRNA (Supplementary Figure S7) and protein levels (Figure 5a). FBLN2 knockdown led to significantly increased tumor cell migration and invasion abilities (Figure 5b). In addition, FBLN2 gene silencing affected the mRNA expression of cell adhesion molecules/regulators and ECM-associated genes with the upregulation of CDH2, ZO2, CX43, DSG2, DSP, DSC3, ITGB4, Fibronectin, Keratin, COL1A1 and MMP14, and with the downregulation of ITGA6 (Figure 5c). Moreover, FBLN2 knockdown increased the levels of phosphorylated ERK1/2 and phosphorylated AKT (Figure 5b), but the level of phosphorylated mTOR did not change. These results, together with those from the FBLN2 transfection, indicate the tumor suppressive role of FBLN2 in NSCLC cells.

### 2.6. FBLN2 Modulates miRNAs in Regulation of Cell Adhesion and ECM Molecules

The dysregulation of miRNAs, including exosome-derived miRNAs has been recognized as a key contributor to lung cancer development, through its integration with gene regulatory networks and interaction with the tumor microenvironment (TME) [28,29]. As cell adhesion mediators/regulators, miRNAs play an important role in cell–cell and cell–matrix interactions. Certain miRNAs have been reported to target cell adhesion molecules [23].

**Figure 6:**
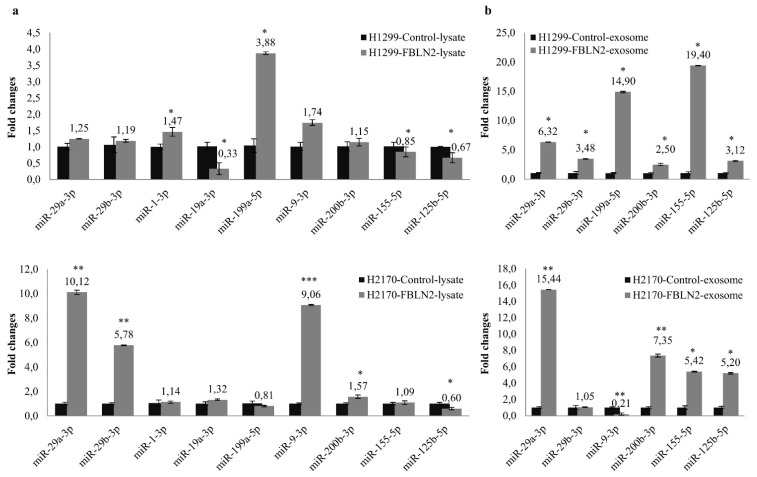
Effect of FBLN2 on both cell lysate and exosome-derived miRNAs. (**a**) Nine miRNAs were widely expressed in cell lysates of both H1299 and H2170 cells, but in different expression patterns. Compared to mock transfectants, miR-29a-3p, miR-29b-3p, miR-9-3p, miR-200b-3p, and miR-125b-5p were highly expressed in H2170 FBLN2 transfectants, while miR-1-3p, miR-199-5p, miR-125b-5p, and miR-21-5p were upregulated in H1299 transfectants. miRNA expression level normalized to U6 in the mock cells was set to 1.0. * *p* < 0.05, ** *p* < 0.01, *** *p* < 0.001. (**b**) In the analysis of exosome-derived miRNA, FBLN2 overexpression upregulated the expression of miR-29a-3p, miR-200b-3p, miR-155b-5p, and miR-125b-5p in both cell lines, and the expression of miR-29b-3p and miR-21-5p in H1299. miRNA expression level normalized to cel-miR-39 in the mock cells was set to 1.0., * *p* < 0.05, ** *p* < 0.01.

To determine whether the ectopic expression of FBLN2 has an effect on miRNAs expression, nine miRNAs involved in the regulation of cell adhesion and ECM composition were analysed in both cell lysates and exosomes isolated from FBLN2 transfected cells (Supplementary Figure S8). The isolated exosomes were characterized using Western blotting with exosome markers CD63 and CD9. High expression of CD63 and CD9 was detected in the exosomes (Supplementary Figure S9). As shown in Figure 6a, miR-1-3p and miR-199-5p were significantly upregulated, while miR-19a-3p, miR-155-5p and miR-125b-5p were downregulated in H1299 cells transfected with FBLN2. Further, in the H2170 cells transfected with FBLN2, miR-29a-3p, miR-29b-3p, miR-9-3p, and miR-200b-3p were markedly upregulated, while miR-125b-5p was downregulated. Regarding the expression levels of the exosomal miRNAs, we found that the FBLN2-overexpressed H1299 cells secreted significantly more miR-29a-3p, miR-29b-3p, miR-199a-5p, miR-200b-3p, miR-155-5p, and miR-125b-5p in H2199, while more miRNA-29a-3p, miR-200b-3p, miR-155-5p, and miR-125b-5p were secreted in H2170 (Figure 6b). In addition, a significantly decreased exosomal miR-9-3p level was observed in H2170 after FBLN2 overexpression (Figure 6b). Like the diverse gene expression pattern between ADC and SCC cells, miRNA expression also varies in ADC and SCC after FBLN2 overexpression, implying that FBLN2 regulates the expression of miRNAs that are related to cell adhesion and ECM composition in a cell type dependent manner.

## 3. Discussion

FBLNs, which are extracellular glycoproteins, interact with cell adhesion molecules and ECM components, and are essential for elastic fiber assembly, ECM stiffness maintaince, and other biological processes [2]. Abnormal expression of FBLNs is associated with clinical pathologies including tumor initiation, progression, and development [1].

As a FBLN family member, FBLN2 has been identified as one of the potential downstream target genes of the tumor suppressor HOPX [30,31]. FBLN2 has been to be upregulated more than 8-fold in HOPX overexpressing cells, compared to mock cells (Supplementary Table S1, [32]). It was reported that FBLN2 exerts a tumor suppressive function in nasopharyngeal cancer (NPC) [15], breast cancer [33] and gastric cancer [19]. Nevertheless, FBLN2 plays an oncogenic role in head and neck squamous cell carcinoma (HNSCC) [34] and murine lung adenocarcinoma [22]. In this study, with the goal of understanding how FBLN2 functions in human lung NSCLC cells, we began the characterization of FBLN2 in human NSCLC.

FBLN2′s expression pattern indicated that it was downregulated in a majority of lung cancer cell lines compared to normal cell line and the short isoform was found to be the main expression form in lung cells, consistent with a previous report [17]. In human normal lung tissues, FBLN2 was highly expressed in bronchial epithelial cells and stroma, indicating its involvement in normal lung development. In primary lung cancer tissues, 44.2% (46/104) of tumor samples exhibited negative staining of the Fibulin 2 protein, and SCC samples displayed significantly higher expression of FBLN2 than ADC, indicating its potential diagnostic value for distinguishing lung SCC from ADC. Additionally, samples with lower T stage tumors tended to have higher FBLN2 expression, providing clues to its possible tumor suppressive function in lung cancer. Similarly, Ibrahim et al. observed that higher FBLN2 expression was associated with favorable outcomes in lymph node negative (lower N stage) breast cancer patients [8].

It is widely accepted that DNA methylation is an important epigenetic mechanism contributing to cancer initiation, maintenance, and progression [35]. Besides genetic alterations, epigenetic regulation has been considered as a secondary mechanism responsible for the inactivation of tumor suppressors [36]. Our data showed that FBLN2 DNA was methylated in the promoter region in most of the lung cancer cell lines displaying downregulated FBLN2, implying that DNA methylation is a frequent epigenetic event partially responsible for FBLN2 gene silencing. In primary lung tumors, DNA methylation was found in 30 out of 37 samples, but statistical analysis did not show a significant correlation between DNA methylation and Fibulin 2 protein expression, probably due to the small sample size. In addition to DNA methylation, histone acetylation might also play a role in the downregulation of FBLN2, since enhanced levels of FBLN2 were also detected in six out of nine lung cancer cell lines after treatment with TAS, a histone deacetylase inhibitor. In fact, the epigenetic regulation of FBLN2 with relation to tumor progression has been depicted in other types of human cancers, including childhood acute lymphoblastic leukemia [37] and colon [38], prostate, breast [26] and nasopharyngeal cancer [15].

The cell-based functional assays showed that the ectopic expression of FBLN2 led to significantly decreased cell proliferation, migration and invasion of both lung ADC (H1299) and SCC (H2170) cells, while siRNA knockdown of FBLN2 in lung ADC cells (H1650) with the endogenous expression of FBLN2 resulted in an enhanced migratory and invasive property. These data together indicate a tumor suppressive function of FBLN2 in human NSCLC cells. The results were not consistence with a previous report by Barid et al. [22], in which an oncogenic function of fbln2 was observed in murine lung ADC cells. However, it is not surprising that FBLN2 exerts different functions in human and murine cancer cells. On the one hand, murine tumor cells cannot fully mimic human tumor cells [39,40], and on the other hand, even in human cancer, FBLN2 may play a dual role, dependent on cell type and context [1,41].

We further investigated the molecular mechanisms underlying the growth inhibitory effects of FBLN2 in NSCLC cells. Overexpression of FBLM2 resulted in decreased activities of MAPK/ERK and AKT/mTOR pathways in both ADC and SCC cells, while knockdown of FBLN2 largely increased the phosphorylated level of AKT and slightly upregulated the phosphorylated level of ERK, but the phosphorylated mTOR did not alter. It is possible due to the unique gene spectrum as well as regulatory networks in H1650. But we could speculate that alteration of AKT and ERK pathway might be sufficient to influence the lung cancer cell migratory and invasive behavior. It is evident that AKT and MPAK signaling pathways play a key role in a wide range of biological processes, including cell differentiation, proliferation, and apoptosis [42,43]. Dysregulation of MAPK and AKT pathways by FBLN2 or its family members has been reported in various types of cancer [41]. Senapati et al. observed that FBLN2 interacted with MUC4–NIDO domain in the basement membrane to promote pancreatic tumor progression via activation of the MAPK pathway [18]. FBLN3 suppresses tumor invasiveness through inhibition of the MAPK pathway in lung cancer [44], and it enhances tumor cell growth and induces epithelial–mesenchymal transition (EMT) by activation of the AKT/mTOR pathway in cervical cancer [45]. Our data indicate that FBLN2-mediated cell inhibitory effects are associated with the suppression of the MAPK and AKT pathways in NSCLC.

As a structural component of elastic fibers, fibulins are closely involved in complex biological functions such as the organization of the extracellular matrix, cell-cell adhesion, and tissue morphogenesis [1]. Recently, a growing body of evidence has pointed out that FLBN2 influences tumor cell behavior by the stimulation or inhibition of cell adhesion molecules, ECM proteins, and their modulators, such as miRNAs [41]. The loss of FBLN2 contributes to invasion in breast cancer through degradation of the architecture basement [46]. In our study, ectopic FLBN2 expression downregulated the ECM component COL1A1 and the cell adhesion receptor ITGB4 (integrin β4), as well as CDH2 (N-cadherin), one of the mediators of cell–cell adhesion on both the mRNA and protein levels. It is worthy to note that the epithelial cell markers CDH1 (E-cadherin) and DSP (desmoplakin) significantly increased, while the mesenchymal cell markers vimentin and CDH2 (N-cadherin) decreased in the FBLN2-transfected cells compared to mock cells. Additionally, in primary lung tumor tissues, it is shown that Fibulin 2 expression is significantly correlated with the expression of E-cadherin and cytokeratin, suggesting the role of fibulin 2 in mesenchymal-epithelial transition (MET). It has been reported that FBLN3 was able to induce EMT in cervical cancer cells [45] and FBLN4 can promote EMT in osteosarcoma [47]. The mechanism through which FBLN2 induce MET is not yet clear. Thus, the function of FBLN2 and other family members in EMT may warrant further research. Again, the gene expression pattern varies between ADC and SCC, reflecting the morphological diversity of the cells. 

The interplay between cell adhesion/ECM and miRNAs has gained much attention recently. Numerous studies have revealed that more than a handful of miRNAs participate in carcinogenesis by regulating cell adhesion molecules and ECM components. For example, miR-29 targets integrin β1 and α6 and thus inhibits the integrin-mediated oncogenic function in HNSCC [48,49], and it also targets a diverse mRNA coding for ECM genes, including collagens, fibrillin and elastin [50]. The tumor suppressor miR-200 affects EMT and tumor cell metastasis by modulating the cell adhesion molecule E-cadherin and the ECM proteins COL6A1, LAMA5, LAMB2, LAMC2, and Fibronectin [51]. In our study, FBLN2 was found to upregulate miR-29a-3p, miR-29b-3p, miR-9-3p, and miR-200-3p in H1299, as well as miR-1-3p and miR-199-5p in H2170. All these selected miRNAs are involved in cell adhesion and ECM construction [23,24,52]. Exosomal miRNAs, as one of the cargoes in the exosomes, act as tumor promoters or suppressors to affect the behavior of the components in the tumor microenvironment which is sustained by a vascular network and the extracellular matrix [53,54]. The discrepant miRNA expression between cell lysates and exosomes might have been due to different exosome motifs and miRNA sequences at the 3′-end [55]. Except for the fact that miR-200b-3p [56] and miR-9-5p [57] may exert an oncogenic function, other miRNAs studied here act as tumor suppressors in NSCLC [57,58,59,60,61,62,63]. The clinical relevance of miRNAs in lung cancer diagnosis, prognosis, and therapy prediction warrants further investigation.

Taken together, based on our data, FBLN2 is a tumor suppressor in NSCLC. It suppresses tumor cell proliferation, migration, and invasion through inhibition of the MAPK and AKT pathways and regulation of cell adhesion molecules as well as ECM-associated genes and miRNAs. Anti-adhesion has evolved to a promising therapeutic concept in oncology [64], and recently, an “anti-ECM” strategy using a function-blocking antibody against Fibulin 3 has been proved in treatment of murine glioblastoma [65]. The cancer growth inhibitory role of FBLN2, as revealed in this study, may provide a basis for further exploring its therapeutic value in human lung cancer.

## 4. Materials and Methods

### 4.1. Cell Lines and Drug Treatment

Human bronchial epithelial cells (HBEC), used as normal control cells for the expression and methylation analyses, were obtained from Clonetics (San Diego, CA, USA) and grown in BEG media (Lonza, Walkersville, MD, USA). One small-cell lung cancer cell line (COLO677), seven lung adenocarcinoma cell lines (H1299, H2030, H23, A549, H322, H1650, and H1975) and three squamous cell carcinoma cell lines (H2170, H226, and H157) were obtained from the American Type Culture Collection (ATCC, Rockville, MD, USA) and the German Collection of Microorganisms and Cell Culture (DSMZ, Braunschweig, Germany). Cells were grown in RPMI 1640 medium (PAN-Biotech, Aidenbach, Germany) supplemented with 10% fetal bovine serum (Biochrom AG, Berlin, Germany) and maintained at 37 °C with 5% CO_2_.

Drug treatment with a DNA methyltransferase inhibitor, 5-aza-2′-deoxycytidine (DAC) or a histone deacetylase inhibitor, Trichostatin A (TSA), was conducted as described previously [66]. Cells treated with the drug-dissolved solution were used as control. The working solutions for DAC and TSA were prepared in phosphate buffered saline (PBS) and dimethyl sulfoxide (DMSO), respectively.

### 4.2. RNA Isolation, cDNA Synthesis and Real-Time RT-PCR 

Total RNA was extracted from the cells using the Trizol reagent (Qiagen, Hilden, Germany) according to the manufacturer’s instructions. For analysis of gene expression, 500 nanogram (ng) of total RNA was applied for cDNA synthesis using a QuantiTect Reverse Transcription Kit (Qiagen, Hilden, Germany). Real-time RT-PCR was performed using a FastStart Universal SYBR Green Master Mix (Roche, Mannheim, Germany). We used glyceraldehyde 3-phosphate dehydrogenase (GAPDH) as an internal control. Primer sequences are shown in Supplementary Table S1. 

### 4.3. DNA Methylation Analysis

Bisulfite modification of genomic DNA was performed by using a DNA methylation kit (Zymo Research, Freiburg, Germany) according to the manufacturer’s protocol. Promoter methylation status of FBLN2 was determined by bisulphite sequencing (BS) in cell lines and methylation-specific-PCR (MSP) in primary tumor tissues. 

The schematic profile of the CpG island of FBLN2 DNA (chr3: 13565624-13654922) is shown in Supplementary Figure S10a, according to the DataBase of CpG islands and Analytical Tool (http://dbcat.cgm.ntu.edu.tw/, accessed on 26 October 2021). The first CpG island including the transcription region, transcription start site, and putative promoter was selected for FBLN2 DNA methylation analysis. In total, 91 CpG sites spanning from −280 to +384 base pairs (bp) (chr3: 13565409-13566075) were analyzed. The positions of the primers within the CpG island used for BS and MSP are highlighted in Supplementary Figure S10b. BS and MSP amplification was carried out on the following conditions: 95 °C 15 min; 94 °C 1 min, annealing temperature 30 s, 72 °C 30 s, 35 cycles; 72 °C 10 min. The annealing temperatures are shown in the Supplementary Table S1. The PCR products from BS were sent to LGC Genomics (Berlin, Germany) for direct sequencing.

### 4.4. Patient Samples and Immunohistochemistry (IHC)

Tissue microarrays (TMAs) containing 104 samples from patients with primary lung tumor were constructed [30]. For one tumor sample, two tissue cylinders of 0.6 mm diameter were included in the TMA. For all patients included in this study, neither chemotherapy nor radiotherapy was performed before surgery. Lung tissues with normal histology adjacent to the tumors were used as normal tissues. The ethical approval for the study was issued by the local ethical committee of University Hospital Jena (Nr: 3815-07/13, Jena, Germany).

IHC was carried out as previously described [67]. IHC was scored semi-quantitatively as score 0 (<10% positively stained cells), score 1 (10–25% positively stained cells), score 2 (25–50% positively stained cells), and score 3 (>50% positively stained cells). For statistical analysis, score 0 was sorted as negative, while scores 1–3 together were considered as positive.

### 4.5. FBLN2 Expression Vector Construction, Stable Transfection, and siRNA Knockdown

The expression vector with the full-length cDNA of the FBLN2 short form was kindly gifted by Prof. Lung (Laboratory of Cancer Molecular Genomics, University of Hong Kong, Pok Fu Lam, Hong Kong). It was cloned into pcDNA3.1/V5-HisB (Invitrogen, Carlsbad, CA, USA) using primers containing Hind III and EcoR I (Supplementary Table S1), and transfected into H2170 and H1299 using transfection reagent Lipofectamine^TM^ 2000 (Invitrogen, Waltham, MA, USA) according to the manufacturer’s protocol. Cells transfected with the empty vector were used as control. Stable transfectants were selected with 400 µg/mL G418 (Santa Cruz Biotechnology, Dallas, TX, USA). Small interfering RNA transient transfection was performed in H1650 with human siRNA-FBLN2 (sc-43119, Santa Cruz Biotechnology, Dallas, TX, USA) and with the control siRNA (sc-37007) using the transfection reagent LipofectamineTM 2000. After 48 h, the cells were collected for RNA/protein expression analysis. 

### 4.6. Colony Formation, Migration, and Invasion

Cells with a total number of 400 were seeded in 6-well plates. After incubation for 10–12 days at 37 °C, the cells were fixed with 100% methanol and stained with 0.5% crystal violet for 10 min. After the plates were air-dried, colonies were defined and counted [68].

For the migration and invasion assays, 3 × 10^4^ cells were suspended in 500 μL medium without FBS and placed in the upper chamber (8 μm pore size, BD Biosciences, Heidelberg, Germany). The upper chamber was placed in a 24-well plate with 750 μL medium containing 20% FBS. After incubation for 48 h, non-migrated cells were removed by a cotton swab, and the migrated cells were fixed and stained in the same way depicted in the colony formation assay. The number of migrated cells was counted in six randomly selected fields. Matrigel-coated transwell chambers (BD Biosciences, Heidelberg, Germany) were applied for the invasion assay [32]. Cells transfected with the empty vector were used as control.

### 4.7. Western Blot (WB) Analysis 

Proteins from the cell lysates were isolated using radioimmunoprecipitation assay buffer (Sigma Aldrich, St. Louis, MO, USA) supplemented with a cocktail of protease inhibitor (Roche), and the protein concentration was determined using a BCA kit (Thermo Scientific, Carlsbad, CA, USA) according to the manufacturer’s protocol. Protein samples (20–35 ng) with loading buffer were loaded into the SDS-polyacrylamide gel, followed by their boiling for 5 min at 95 °C. After the electrophoresis and transferation of the proteins on 0.2 µm nitrocellulose membranes (GE Healthcare, Munich, Germany), they were then blocked for 1 h and incubated with primary antibodies overnight. After washing them three times with TBST, the membranes were incubated with secondary antibodies for 1 h and washed again. Enhanced chemiluminescence (Santa Cruz Biotechnology, Dallas, TX, USA) was used for signal detection. Information about the primary and secondary antibodies applied in this study is shown in Supplementary Table S2.

### 4.8. Exosome Isolation, miRNA Analysis

Cell culture, collection of the cell culture medium, and exosome isolation were carried out as previously described [32]. Isolated exosomes (5 ng) were characterized by WB. For miRNA analysis, 270 ng of total RNA from paired lysates and exosomes were reversely transcribed to cDNA using a miScript II RT Kit (Qiagen, Hilden, Germany), following the manufacturer’s guide. U6 and the Cel-miR-39 Spike-In Kit (Norgen Biotek Corporation, Thorold, ON, Canada) were used as internal controls for the lysate and exosome miRNA analyses, respectively. Real-time RT-PCR was carried out using the miRCURY LNA RT Kit and the miRCURY LNA miRNA SYBR Green PCR Kit (Qiagen, Hilden, Germany). Ten selected miRCURY LNA miRNAs involved in cell adhesion and extracellular matrix remodeling were synthesized by Qiagen (Hilden, Germany).

### 4.9. Statistical Analysis

Statistical analysis was performed using IBM SPSS Statistics 21. The association between Fibulin 2 protein expression, DNA methylation and clinicopathological parameters was analysed using two-tailed chi-square or Fisher’s exact test. Assessment of the relative gene/miRNA expression between FBLN2 transfectants and control cells, or transient transfection groups with siRNA-FBLN2 and the control siRNA, was carried out by two-tailed Student’s t-test after normalization with internal controls (GAPDH, U6, or Cel-miR-39). *p*-values less than 0.05 were considered statistically significant.

## Figures and Tables

**Table 1:** Association between fibulin 2 protein expression and clinicopathological parameters in primary lung tumors.

		Fibulin 2			
		Negative	Positive	*p*-Value	
Type	ADC	28	24		
	SCC	14	28	0.047	
Methylation status	Unmethylation	4	3		Fisher’s
	Partial methylation and methylation	15	15	1	
Gender	Male	29	39		
	Female	14	14	0.51	
Age	≤65	34	42		
	>65	9	11	0.983	
pT	1–2	42	45		Fisher’s
	3–4	2	10	0.061	
pN	0–1	27	39		
	2–4	16	14	0.257	
pM	0	40	49		Fisher’s
	1–3	3	4	1	
Grade	1–2	20	28		
	3–4	23	25	0.538	

ADC, adenocarcinoma; SCC, squamous cell carcinoma. pT: tumor size; pN: tumor invasion in lymph node; pM: tumor metastasis.

**Table 2:** Pearson correlation coefficients between Fibulin 2 and Fibronectin protein expression in primary lung tumors.

Lower T Stage	Fibulin 2 vs. Fibronectin		
	ADC + SCC	ADC	SCC
Sample number	49	23	26
Correlation coefficient (*r* Value)	−0.204	0.016	−0.391
Sig. (2-tailed) *p*	0.16	0.944	0.048

**Table 3:** Pearson correlation coefficients between fibulin 2 and E-cadherin, vimentin and cytokeratin protein expression in primary lung tumors.

	Fibulin 2 vs. E-Cadherin			Fibulin 2 vs. Vimentin			Fibulin 2 vs. Cytokeratin		
	ADC + SCC	ADC	SCC	ADC + SCC	ADC	SCC	ADC + SCC	ADC	SCC
Sample number	81	45	36	79	44	35	78	44	34
Correlation coefficient (*r* Value)	−0.031	−0.206	0.358	−0.105	−0.056	−0.207	0.144	−0.047	0.422
Sig. (2-tailed) *p*	0.781	0.175	0.032	0.357	0.716	0.233	0.208	0.764	0.013

## Data Availability

Data is contained within the article or Supplementary Materials.

## Supplementary Materials

The following are available online at https://www.mdpi.com/article/10.3390/ijms222111834/s1.

## Abbreviations

ECM	Extracellular matrix
EMT	Epithelial–mesenchymal transition
MET	Mesenchymal–epithelial transition
CDH1	E-cadherin
CDH2	N-cadherin
CDH3	P-cadherin
JAM	Junctional adhesion molecule
ZO	Zonula occludens
CX	Connexin 43 (Gap junction alpha-1)
ITGB	Integrin beta
ITGA	Integrin alpha
ELN	Elastin
COL1A1	Collagen Type 1 alpha 1
MMP	Matrix metallopeptidase
TIMP	Metallopeptidase inhibitor
DSP	Desmoplakin
DSG2	Desmoglein 2
DSC2	Desmocollin 3
PKG	Plakoglobin
