# Supplementary material for: Fibulin 2 Is Hypermethylated and Suppresses Tumor Cell Proliferation through Inhibition of Cell Adhesion and Extracellular Matrix Genes in Non-Small Cell Lung Cancer
Source: Int J Mol Sci. 2021 Oct 31;22(21):11834. doi: 10.3390/ijms222111834 (PMC8584407; doi:10.3390/ijms222111834)
Supplement: Supplementary file 1 [file ijms-22-11834-s001.zip › ijms-1423486 supplementary figures.pdf]

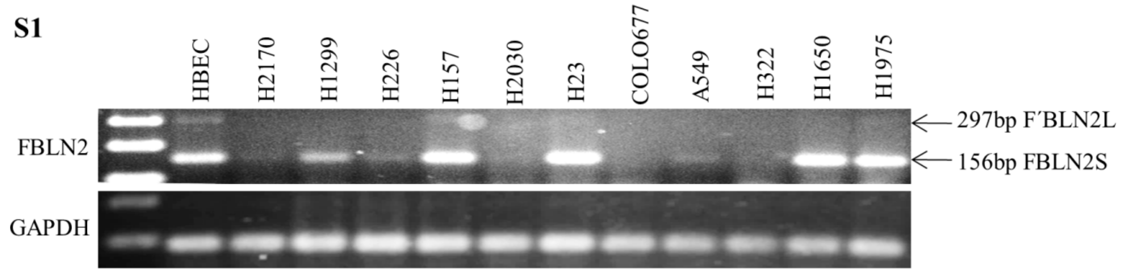

Figure S1. The mRNA expression of FBLN2 long and short isoforms in normal human bronchial epithelial cells and a panel of lung cancer cell lines using RT-PCR analysis.

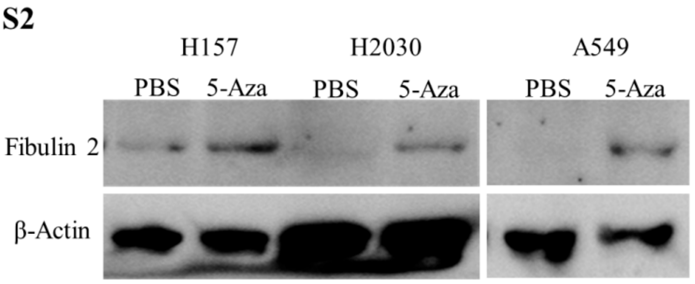

Figure S2. Protein expression of FBLN2 after demethylation treatment with 5 μM of 5-aza-2′-deoxycytidine (DAC) in NSCLC cells H157, H2030, and A549.

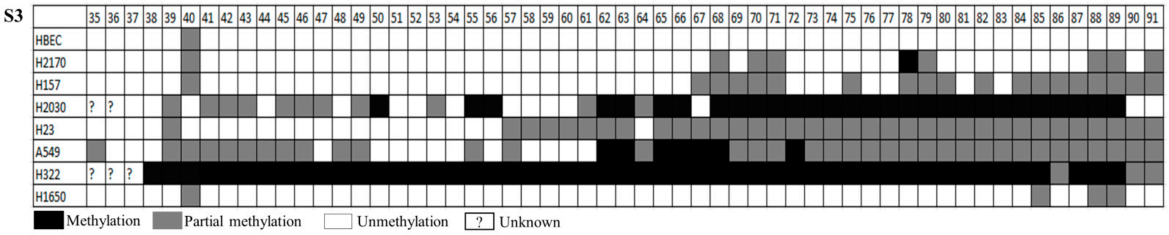

Figure S3. Methylation status of FBLN2 at CpG sites 51–91.

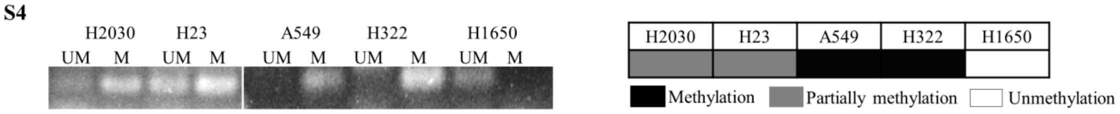

Figure S4. Validation of the primers used for MSP in lung cancer cell lines.

S5

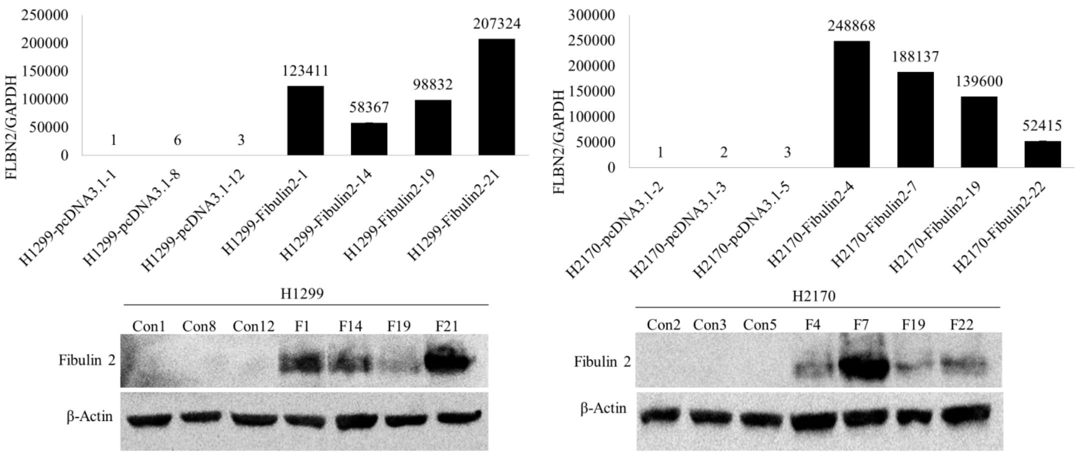

Figure S5. mRNA and protein expression of FBLN2 after stable transfection with the FBLN2 expression vector.

S6

**Cell adhesion molecules**	**ECM associated genes**
Tight junction: JAM1, ZO1/2	Fibronectin, Vimentin, Elastin
Adherent junction: CDH1-3	Keratin
Desmosome: DSP, DSG2, DSC3, PKG	COL1A1, COL3A1, COL4A1
Gap junction: CX26, CX26	MMPs (MMP2, 7, 9, 14)
Hemidesmosome: ITGB4, ITGB1, ITGA6	TIMP1/2 (MMPs inhibitor)

Figure S6. Selected panel of cell adhesion molecules and ECM-associated genes for the expression analysis.

S7

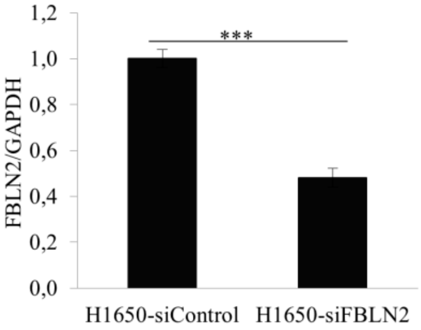

Figure S7. mRNA expression of FBLN2 after siRNA transient transfection in H1650 cells, mRNA expression level compared to GAPDH in cells transfected with siRNA-Control was set to 1.0. *** $p < 0.001$.

S8

	miRNA Name	Cat. No. (Qiagen)
1	miR-29a-3p	YP00204698
2	miR-29b-3p	YP00204679
3	miR-1-3p	YP00204344
4	miR-19a-3p	YP00205862
5	miR-199a-5p	YP00204494
6	miR-9-3p	YP00204620
7	miR-200b-3p	YP00206071
8	miR-155-5p	YP00204308
9	miR-125b-5p	YP00205713
10	U6 snRNA	YP00203907

Figure S8. The list of miRNAs analyzed in this study.

S9

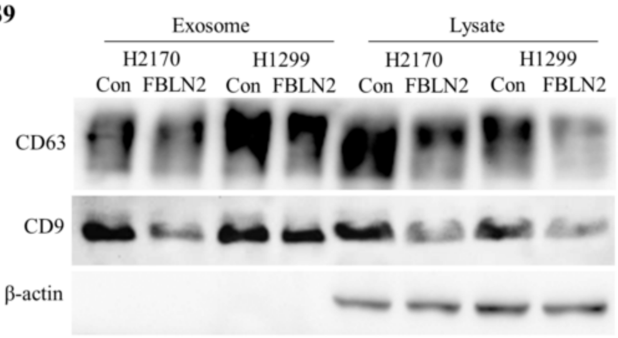

Figure S9. Western blotting of exosome markers CD63 and CD9 in both cell lysates and isolated exosomes of H2170 and H1299 cells. β-actin was used as loading control for proteins isolated from the cell lysates.

S10

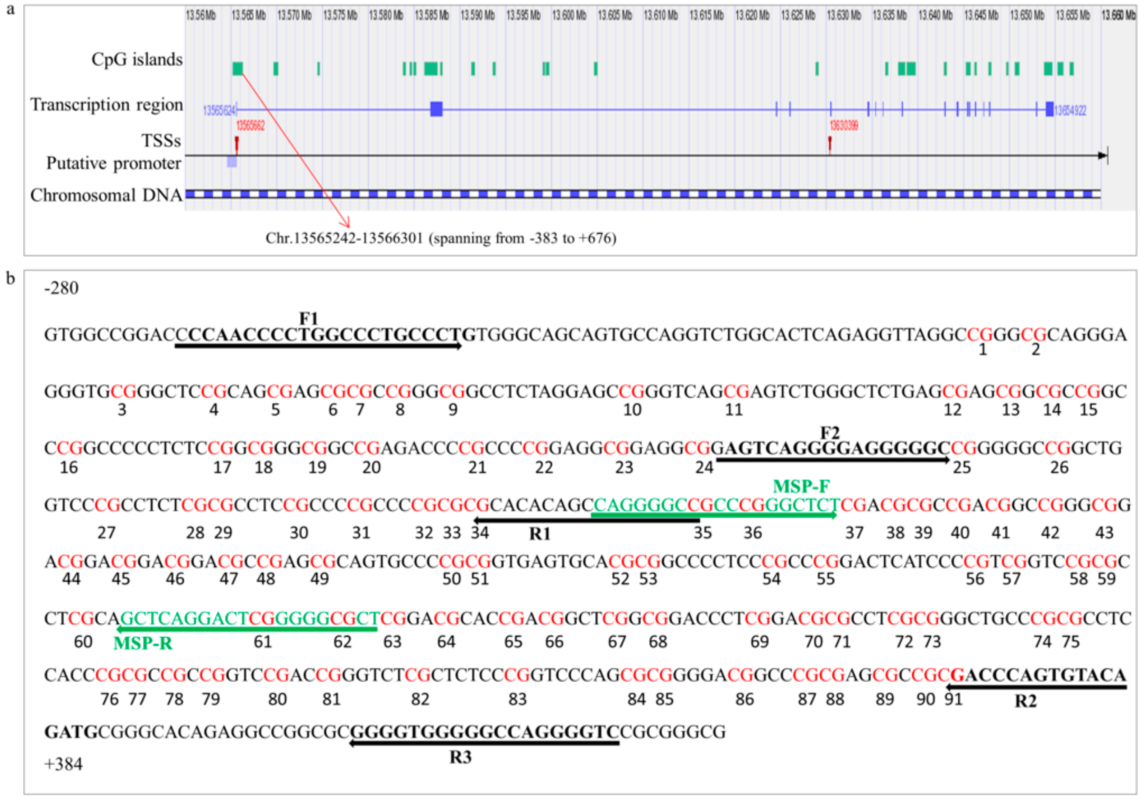

Figure S10. (a) Schematic profile of the FBLN2 CpG islands (http://dbcat.cgm.ntu.edu.tw/). (b) Primers used for BS (black underlined) and MSP (green underlined) are indicated within the CpG island spanning from −280 to +384 bp (Chr.: 13565409-13566075).
