# Supplementary figures and images for: Fibulin 2 Is Hypermethylated and Suppresses Tumor Cell Proliferation through Inhibition of Cell Adhesion and Extracellular Matrix Genes in Non-Small Cell Lung Cancer
Source: Int J Mol Sci. 2021 Oct 31;22(21):11834. doi: 10.3390/ijms222111834 (PMC8584407; doi:10.3390/ijms222111834)

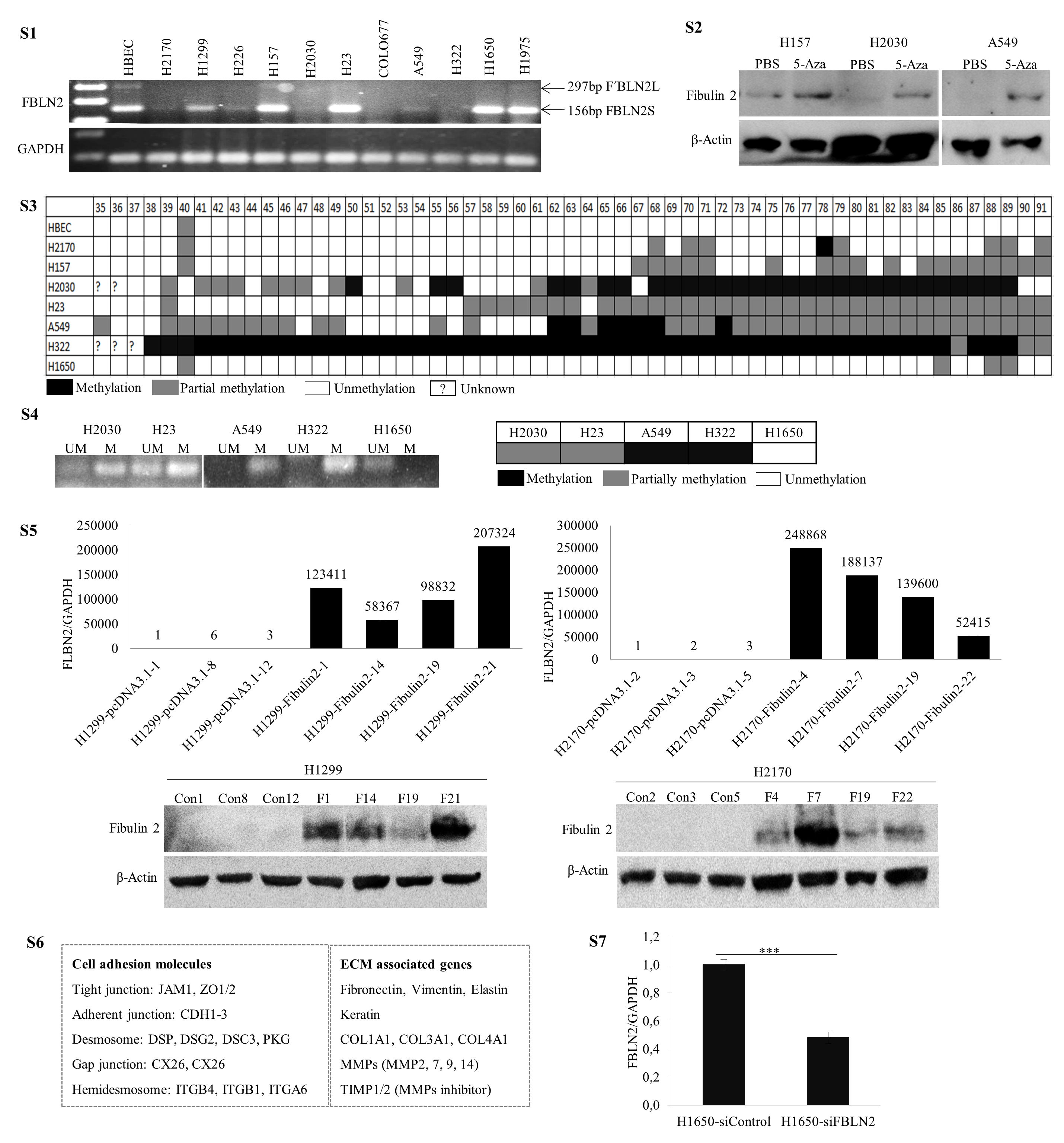

Supplement: Supplementary file 1 [file ijms-22-11834-s001.zip › Supplementary figure 1-7.tif]

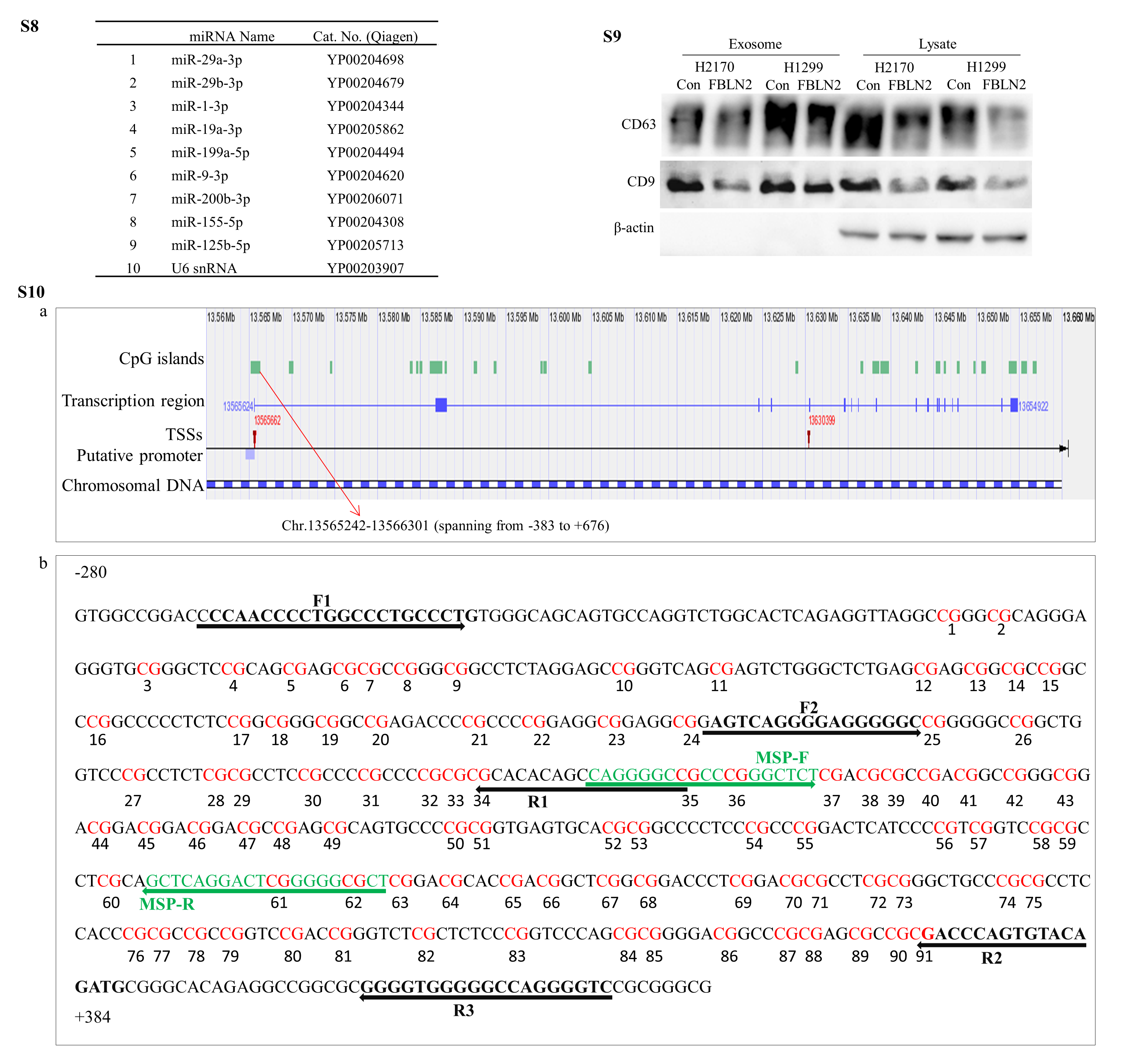

Supplement: Supplementary file 1 [file ijms-22-11834-s001.zip › Supplementary figure 8-10.tif]
